# The Antioxidant Salidroside Ameliorates the Quality of Postovulatory Aged Oocyte and Embryo Development in Mice

**DOI:** 10.3390/antiox13020248

**Published:** 2024-02-19

**Authors:** Kexiong Liu, Luyao Zhang, Xiaoling Xu, Linli Xiao, Junhui Wen, Hanbing Zhang, Shuxin Zhao, Dongliang Qiao, Jiahua Bai, Yan Liu

**Affiliations:** 1 Institute of Animal Husbandry and Veterinary Medicine, Beijing Academy of Agriculture and Forestry Sciences, Beijing 100097, China; liukexiong2023@163.com (K.L.);; 2 Key Laboratory of Adaptation and Evolution of Plateau Biota, Northwest Institute of Plateau Biology, Chinese Academy of Sciences, Xining 810001, China; zhangluyao@nwipb.cas.cn; 3 Development Center of Science and Technology, Ministry of Agriculture and Rural Affairs, Beijing 100176, China

**Keywords:** salidroside, postovulatory aging, oocyte, autophagy, oxidative stress, MAPK

## Abstract

Postovulatory aging is known to impair the oocyte quality and embryo development due to oxidative stress in many different animal models, which reduces the success rate or pregnancy rate in human assisted reproductive technology (ART) and livestock timed artificial insemination (TAI), respectively. Salidroside (SAL), a phenylpropanoid glycoside, has been shown to exert antioxidant and antitumor effects. This study aimed to investigate whether SAL supplementation could delay the postovulatory oocyte aging process by alleviating oxidative stress. Here, we show that SAL supplementation decreases the malformation rate and recovers mitochondrial dysfunction including mitochondrial distribution, mitochondrial membrane potential (ΔΨ) and ATP content in aged oocytes. In addition, SAL treatment alleviates postovulatory aging-caused oxidative stress such as higher reactive oxygen species (ROS) level, lower glutathione (GSH) content and a reduced expression of antioxidant-related genes. Moreover, the cytoplasmic calcium ([Ca^2+^]c) and mitochondrial calcium ([Ca^2+^]mt) of SAL-treated oocytes return to normal levels. Notably, SAL suppresses the aging-induced DNA damage, early apoptosis and improves spindle assembly in aged oocytes, ultimately elevating the embryo developmental rates and embryo quality. Finally, the RNA-seq and confirmatory experience showed that SAL promotes protective autophagy in aged oocytes by activating the MAPK pathway. Taken together, our research suggests that supplementing SAL is an effective and feasible method for preventing postovulatory aging and preserving the oocyte quality, which potentially contributes to improving the successful rate of ART or TAI.

## 1. Introduction

Salidroside (SAL) is a phenylpropanoid glycoside that can be found naturally in the genus *Rhodiola* plant, which originates from the mountains of southwest China and the Himalayas [1]. Numerous studies have demonstrated the wide range of pharmacological effects exhibited by SAL, such as its ability to alleviate hypoxia, combat fatigue, slow down aging processes, inhibit cancer growth, and reduce inflammation [2,3,4,5,6]. Additionally, the positive effects on various diseases have been reported, including cardiovascular and central nervous system [7,8]. Consequently, it is considered to be a potential therapeutic substance for preventing and treating various diseases. Most importantly, salidroside also has a good antioxidant performance. Salidroside has been reported to possess a significant inhibitory effect on oxidative stress induced by diabetes, and its mechanism is correlated with the increased levels of serum insulin and activity of SOD, GPx, and catalase [9]. In 2015, a study found that the contrast agents-induced kidney damage could be significantly prevented and ameliorated through decreasing MDA level and increasing SOD, NO and mRNA and protein levels of eNOS after treatment with salidroside [10]. Two recent studies found that SAL improves pig oocyte maturation and subsequent embryonic development by promoting lipid metabolism and meiotic resumption [11,12]. In addition, SAL alleviates oxidative stress and apoptosis via AMPK/Nrf2 pathway in DHT-induced human granulosa cell line KGN [13].

In mammalian species, the occurrence of successful fertilization requires the optimal fertilization window. Moreover, estrus marks the time of ovulation, which is consistent with mating time, allowing sperm and oocytes in the fallopian tubes to meet simultaneously. Following ovulation, female oocytes are arrested at the metaphase of the second meiosis (MII) and remain receptive to fertilization by spermatozoa. This can occur either within the oviduct or in an artificial culture setting, with a time limit of 12 h for rodents and 24 h for monkeys and humans. Failure to achieve fertilization within these timeframes leads to a time-dependent breakdown process termed postovulatory aging (POA) [14]. Compared with fresh oocytes, POA oocytes would exhibit various oocyte defects, including morphological, molecular, genomic, and epigenetic anomalies, that could lead to oocyte apoptosis [15,16,17,18,19,20,21]. Hence, the significance of POA in human-assisted reproduction cannot be overlooked, as it may lead to the postponement of fertilization for retrieved mature oocytes owing to heightened burdens on laboratory operations and delays in acquiring semen samples. In the livestock industry, the simultaneous estrus-timed insemination technique was widely applied. But in this process, some female animals cannot undergo timely insemination after estrus or ovulation, resulting in lower pregnancy rates.

The potential mechanisms of postovulatory aging have not yet been revealed. However, numerous studies have indicated a close association between oxidative stress and the deterioration of quality in POA oocytes [20]. The research indicated that postovulatory aging causes DNA damage and apoptosis, abnormal levels of ROS and GSH, and mitochondrial dysfunction, etc. in oocytes, meanwhile, the SIRT and NRF2 pathways may be involved in this process [15,22]. Hence, the addition of antioxidants has the potential to function as a secure and efficient approach to postpone or hinder the aging process of oocytes via the mitigation of oxidative stress. Within the last five years, many antioxidants have been used to delay postovulatory oocyte aging in mice or pigs [23,24]. During the action of these antioxidants, the increase in ROS production and the mitochondrial dysfunction were significantly improved. Nevertheless, the protective role of SAL during oocyte aging and its underlying mechanism still lack clarity.

In the present study, we exploit an in vitro aging model to explore the effects of SAL on postovulatory aged oocytes in mice. Our findings suggest that the supplementation of SAL can effectively reverse the decline in oocyte quality caused by aging, enhancing the ability of early embryonic development. In addition, we further determined that SAL treatment promotes protective autophagy to ameliorate quality deterioration of POA oocytes by activating the MAPK pathway.

## 2. Materials and Methods

### 2.1. Chemicals and Animals

All chemicals were purchased from Sigma–Aldrich (St. Louis, MO, USA) unless specifically stated. CD-1^®^ (ICR) mice were bought from the Beijing Vital River Experimental Animals Centre (Beijing, China) and were kept in cages with proper ventilation, following a 12-h light and 12-h dark routine (lights on from 08:00 to 20:00), while maintaining a controlled temperature of 22 ± 2 °C. A total of 40 mice were used in this study; they had access to food and water without any limitations.

### 2.2. Oocyte Collection and Parthenogenesis Activation

Mice were injected with 10 IU PMSG, followed by 10 IU human chorionic gonadotrophin (hCG, Ningbo Sansheng Pharmaceutical, Ningbo, China) 48 h later to collect MII oocytes. At 13–14 h post-hCG injection, MII oocytes were collected from the oviduct ampulla and collected in M2 medium, the cumulus cells were removed using 0.1% (*w*/*v*) hyaluronidase. Meanwhile, Different concentrations of SAL (0, 10, 20, 40 μM) were added into the medium and treated at different times (6, 12, 18, 24 h). For parthenogenesis activation, denuded oocytes were transferred first into (Ca^2+^)-free human tubal fluid (HTF) medium contained with 10 mmol/L strontium chloride and 5 μg/mL cytochalasin B (Merck, Darmstadt, Germany), cultured at 37 °C with 5% CO_2_ for 2.5 h. Then oocytes were transferred into HTF with 5 μg/mL cytochalasin B, incubated at 37 °C with 5% CO_2_ for 3.5 h. Activated oocytes were then cultured in KSOM plus (+) amino acids (KSOM/AA) medium (EmbryoMax^®^ KSOM + AA with D-glucose and phenol red, EMD Millipore, Billerica, MA, USA) at 37 °C with 5% CO_2_ for early embryo development. Different stages of embryonic development (2-cell, 4-cell, morula and blastocyst) were recorded at 24, 48, 96, and 120 h, respectively.

### 2.3. Immunofluorescence (IF) Staining and Confocal Microscopy

Mouse oocytes/embryos were fixed in 4% (*w*/*v*) paraformaldehyde (PFA) for 40 min, followed by permeabilization with 0.1% Triton X-100 for 1 h. After 1 h of blocking in the blocking buffer (1% BSA/PHEM with 100 mM glycine), the samples were incubated with different primary antibodies (anti-γH2A.X, 1:100; anti-α-Tubulin, 1:8000; anti-BAX, 1:100; anti-Cleaved caspase 3, 1:100; anti-BECN1, 1:100; anti-LC3B, 1:100; anti-p-MAPK, 1:50) overnight at 4 °C. The samples were incubated with a secondary antibody for 1 h at room temperature. Finally, all samples were stained with Hoest33342 (Sangon Biotech, Co., Ltd., Shanghai, China). The fluorescent images were taken with laser scanning confocal microscopy (A1 Cell Imaging System; Nikon, Tokyo, Japan) with the same parameters.

### 2.4. Quantification of [Ca^2+^]c and [Ca^2+^]mt

Levels of cytoplasmic calcium ([Ca^2+^]c) and mitochondrial calcium ([Ca^2+^]mt) were measured using Fluo 4-AM and Rhod 2-AM (Invitrogen/Molecular Probes, Carlsbad, CA, USA) staining, respectively. The zona pellucida was removed using 0.5% pronase E, then oocytes were incubated with dye for 30 min at 37 °C with 5% CO_2_. Oocytes were washed with DPBS three times and observed by confocal laser scanning microscopy (Nikon A1R, Tokyo, Japan) and quantified using a NIS-Elements AR (Nikon Instruments, Tokyo, Japan).

### 2.5. Mitochondrial Density and Mitochondrial Membrane Potential

To assess mitochondrial density, oocytes underwent incubation in M2 medium contained with a 5 μmol/L Mito-Tracker (red) dye (Beyotime, Shanghai, China) for 30 min. Subsequently, the oocytes were subjected to triple washes with DPBS followed by analysis utilizing a confocal laser scanning microscope. For the evaluation of mitochondrial membrane potential (MMP), oocytes were examined using the JC-1 assay kit (Beyotime, Shanghai, China). In brief, oocytes were randomly assigned to three groups for different treatments. Following, all oocytes were treated with a staining solution comprising 10 mmol/L JC-1 and maintained at a temperature of 37 °C with a CO_2_ concentration of 5% for 20 min. Thereafter, the oocytes were washed three times with DPBS and visualized using a laser scanning confocal microscope within the same z-stacks (Nikon A1R, Tokyo, Japan). All detection operations on the three groups of oocytes were performed simultaneously. The healthy mitochondria show a higher MMP calculated as the ratio of red fluorescence to green fluorescence.

### 2.6. ATP Content Assays

The ATP content in each oocyte was measured using the Enhanced ATP Assay Kit (Beyotime, Shanghai, China) according to the manufacturer’s instructions. Briefly, different ATP standards were prepared (from 0 to 40 pmol). Oocytes were then treated with 20 μM of lysis buffer and lysed cells were centrifuged for 5 min at 4 °C. ATP detection diluent, standard solutions, and ATP assay buffer were injected into 96-well plates, and luminescence signals were promptly measured using a luminometer (Infinite F200; Tecan, Männedorf, Zürich, Switzerland). The standard curve was employed for the calculation of ATP content. The average content per oocyte (pmol/oocyte) was determined by dividing the total ATP amount by the number of oocytes in each sample.

### 2.7. Annexin-V Staining of Oocytes

Annexin-V is recognized as one of the sensitive indicators for the detection of early apoptosis. Therefore, the Annexin-V staining kit (Vazyme, Nanjing, China) was utilized to detect the apoptotic state of different treated oocytes following the instructions provided by the manufacturer. In short, the oocytes were treated with 100 μL of binding buffer and 5 μL of Annexin-V-FITC, then incubated at 37 °C for 10 min. The fluorescent signals were analyzed by a fluorescence microscope (IX73, Olympus, Tokyo, Japan).

### 2.8. Measurement of Intracellular Levels of ROS and GSH

The denuded oocytes were incubated in the medium containing 1 mmol/L 2′,7′-dichlorofluorescein diacetate (2′,7′-DCFHDA) for measuring ROS or 10 μmol/L CellTracker Blue (Invitrogen, Carlsbad, CA, USA) for measuring GSH for 30 min at 37 °C in 5% CO_2_. Then oocytes were washed with DPBS three times. The fluorescence was measured under a fluorescence microscope (DP72, Olympus, Tokyo, Japan) with a filter at 460 nm excitation for ROS and 370 nm excitation for GSH. The fluorescence of each oocyte was determined by EZ-C1Free-Viewer (Nikon, Tokyo, Japan).

### 2.9. Quantitative Real-Time-PCR (qRT-PCR)

Total RNA was extracted from MII oocytes/embryos using the TRNzol Reagent (Tiangen Biotech, Beijing, China) and was reverse transcribed using the FastQuant RT Kit (Tiangen Biotech) following the standard protocol. The qPCR reaction was performed using SuperReal PreMix Kit (Tiangen Biotech) on an ABI 7500 system (Applied Biosystems). The cycle threshold (CT) value of genes was obtained from three replicates. Relative mRNA levels of target genes were calculated using the 2^−ΔΔCt^ method. The primers used are presented in Table 1.

### 2.10. RNA-Seq

The zona pellucida of oocytes was removed using 0.5% pronase E. Then, denuded oocytes were washed with DPBS three times, transferred to cell lysis buffer containing RNase inhibitor, and stored at −80 °C. After cDNA amplification by Smart-Seq2, the RNA-seq was carried out by Annoroad Gene Tech. Co., Ltd. (Beijing, China). Briefly, the cDNA samples were purified using Beckman AMPure XP, and their concentration was identified by NanoPhotometer^®^ (IMPLEN, Munich, Germany). RNA concentration and integrity were detected by Agilent 2100 RNA nano 6000 assay kit (Agilent Technologies, SCC, CA, USA). Sequencing libraries were generated using VAHTS Universal V6 RNA-seq Library Prep Kit for Illumina^®^ (NR604-01/02) following the manufacturer’s recommendations. Libraries were sequenced on an Illumina platform and 150 bp paired-end reads were generated. The cluster generation and sequencing were performed on the Novaseq 6000 S4 platform. Reads were mapped to the reference genome using HISAT2. Quantification of gene expression was performed using HTSeq version 0.6.0. Differentially expressed genes (DEGs) were defined as those with |log2 Fold change| ≥ 1 and an adjusted *p* value < 0.05. The GOseq R packages and KOBAS software 3.0 were utilized to execute the GO enrichment and KEGG pathway enrichment analyses, respectively.

### 2.11. Statistical Analysis

Each experiment was repeated at least three times. Statistical analysis was performed using the SPSS 24.0 Software. All percentages or values were expressed as mean ± SEM. Data were analyzed by analysis of variance (ANOVA) followed by post hoc analysis and Student’s *t*-test. The level of significance was accepted as ns *p* > 0.05 * *p* < 0.05; ** *p* < 0.01; *** *p* < 0.001.

## 3. Results

### 3.1. SAL Decreases Oocyte Malformation Rate during Postovulatory Aging

**Figure 1:**
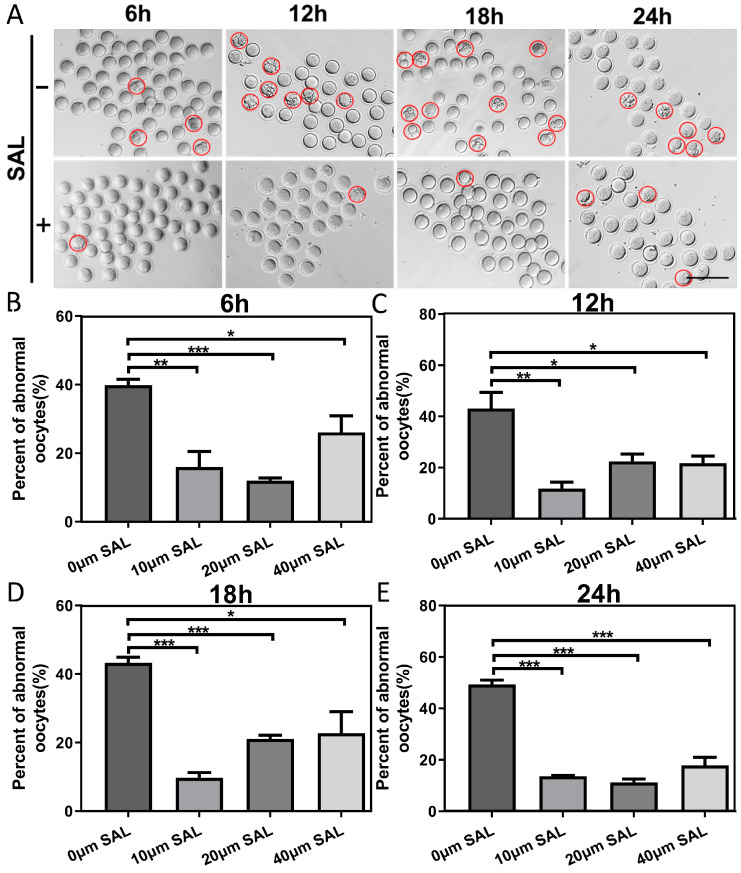
SAL decreases oocyte malformation rate during postovulatory aging. (**A**) Representative images of oocytes aging 6, 12, 18, and 24 h and the effect of SAL treatment (scale bar = 150 μm); the red circles represent abnormal oocytes. (**B**–**E**) The rate of abnormal oocytes treated with 0, 10, 20, and 40 μM SAL after aging 6, 12, 18, and 24 h. Results are presented as mean ± SEM. * *p* < 0.05; ** *p* < 0.01; *** *p* < 0.001.

To investigate the potential effect of SAL in the improvement of the quality of postovulatory aged oocytes, we treated oocytes with different concentrations of SAL (0, 10, 20, 40 μM). Then, we examined the morphology and integrity of ovulated mouse oocytes aged for 6, 12, 18, and 24 h in vitro following SAL supplementation. The occurrence of fragmentation was observed and calculated. As shown in Figure 1A and Table S1, a higher frequency of fragmented oocytes was present in the aged group at different times after ovulation. The quantitative results showed that the fragmentation rate of oocytes in the aged group decreased significantly after all concentrations of SAL treatment especially 10 μM and 20 μM (Figure 1B–E). In subsequent experiments, we cultured ovulated oocytes for 6 h and treated them with 10 um SAL for further research.

### 3.2. SAL Recovers Oocyte Mitochondrial Dysfunction during Postovulatory Aging

**Figure 2:**
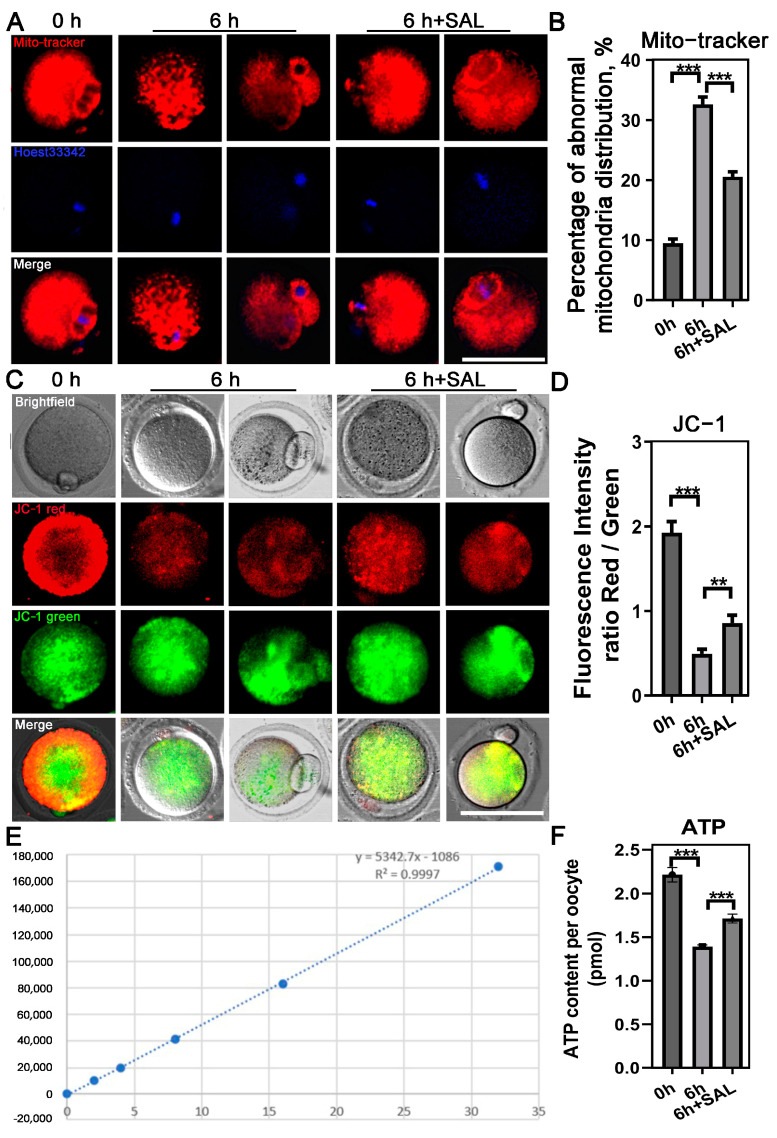
SAL recovers mitochondrial dysfunction during postovulatory aging. (**A**) Representative images of mitochondrial distribution in oocytes. (**B**) Rate of abnormal mitochondria distribution (0 h n = 38; 6 h n = 31; 6 h + SAL n = 38). (**C**) Representative images of mitochondrial ΔΨm in oocytes. (**D**) Quantification of the mitochondrial ΔΨm (0 h n = 37; 6 h n = 23; 6 h + SAL n = 29). (**E**) The standard curve for the detection of ATP content. (**F**) Quantification of the ATP level (0 h n = 40; 6 h n = 40; 6 h + SAL n = 40). Scale bar = 50 μm. Results are presented as mean ± SEM. ** *p* < 0.01; *** *p* < 0.001.

Mitochondrial dynamics, strongly linked to oxidative stress, plays a crucial role in ATP generation in oocytes and embryos. Consequently, we examined the functionality and performance of mitochondria after an extended period of postovulatory aging by assessing their localization within the cell and mitochondrial membrane potential (ΔΨm). Normal ovulated oocytes exhibited a uniform dispersion throughout the cytoplasm, condensing mainly around the chromosomes. In contrast, aged ova displayed an uneven distribution, characterized by large aggregations in the cell’s cytoplasm. These aberrant distribution patterns were significantly alleviated upon SAL treatment in the 6-h experimental groups (0 h vs. 6 h, *p* < 0.001; 6 h vs. 6 h + SAL, *p* < 0.001; Figure 2A,B).

The abnormal distribution of mitochondria suggests that their function may be impaired in aging oocytes. To verify it, we first tested the MMP (ΔΨm) by JC-1 staining. The quantitative analysis revealed that the ratio of red and green fluorescence was significantly lower in aged oocytes than that in control but rescued in SAL-treated oocytes (0 h vs. 6 h, *p* < 0.001; 6 h vs. 6 h + SAL, *p* < 0.01; Figure 2C,D). Since one of the most important functions of mitochondria is to produce ATP for cell development, we additionally measured the ATP content in each group. We observed that ATP levels were dramatically reduced with aging, but increased upon SAL treatment (0 h vs. 6 h, *p* < 0.001; 6 h vs. 6 h + SAL, *p* < 0.001; Figure 2E,F). These results indicate that SAL supplementation could prevent the aging-caused mitochondrial dysfunction in oocytes.

### 3.3. SAL Alleviates Oxidative Stress in Aged Oocytes

**Figure 3:**
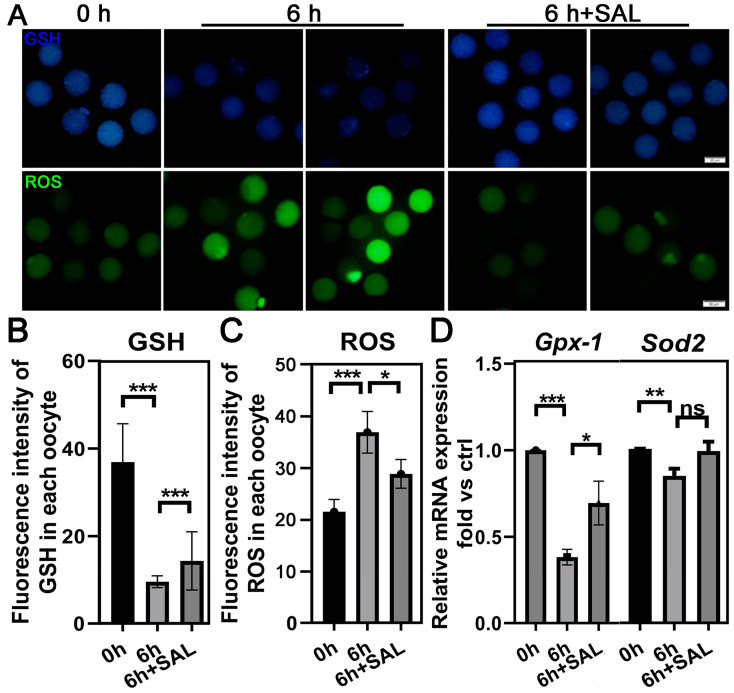
SAL recovers redox status during postovulatory aging. (**A**) Representative images of GSH and ROS levels in young and aged oocytes with or without SAL administration (scale bar = 50 μm). (**B**) The fluorescence level of GSH signals (0 h n = 36; 6 h n = 27; 6 h + SAL n = 37). (**C**) The fluorescence level of ROS signals (0 h n = 38; 6 h n = 30; 6 h + SAL n = 33). (**D**) The mRNA expression of *Gpx-1* and *Sod2* in MII oocytes. Results are presented as mean ± SEM. ns *p* > 0.05; * *p* < 0.05; ** *p* < 0.01; *** *p* < 0.001.

Oxidative stress has been considered a crucial mechanism underlying cellular aging. To assess the effect of SAL on protecting oocytes from oxidative stress during postovulatory aging, we measured the levels of intracellular ROS and GSH level in oocytes with CellTracker Blue and DCFH-DA, respectively. As shown in Figure 3A,B, the intracellular GSH level in aged oocytes was significantly lower than that in control oocytes, but SAL treatment significantly increased the GSH content in aged oocytes (0 h vs. 6 h, *p* < 0.001; 6 h vs. 6 h + SAL, *p* < 0.001). On the other hand, postovulatory aging causes ROS accumulation in oocytes, while decreased by SAL treatment (0 h vs. 6 h, *p* < 0.001; 6 h vs. 6 h + SAL, *p* < 0.05; Figure 3A,C). Finally, we determined the mRNA expression of antioxidant and oxidative stress related genes (*Gpx-1* and *Sod-2*) by qRT-PCR analysis. Compared to the ovulated oocytes, the aged oocytes showed significantly decreased mRNA levels of *Gpx-1* and *Sod-2*; however, the mRNA levels of these genes were significantly increased after SAL treatment (Figure 3D). Altogether, these observations indicated that SAL could reverse the oxidative damage caused by ROS accumulation and the lack of GSH during postovulatory aging.

### 3.4. SAL Restores [Ca^2+^]c and [Ca^2+^]mt Levels in Aged Oocytes

**Figure 4:**
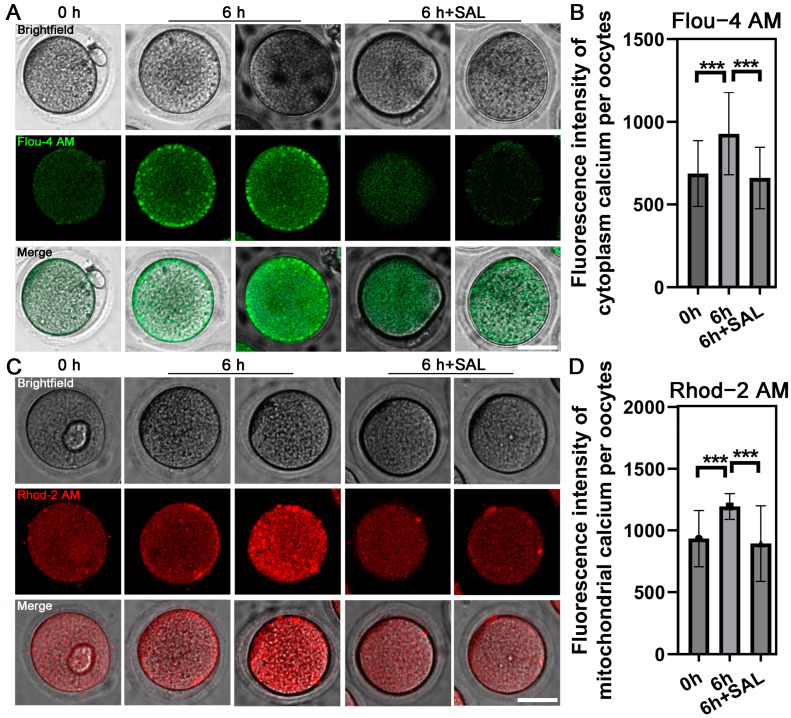
SAL recovers calcium levels during postovulatory aging. (**A**) Representative images of [Ca^2+^]c in three groups. (**B**) Quantification of the [Ca^2+^]c level (0 h n = 34; 6 h n = 38; 6 h + SAL n = 25). (**C**) Representative images of [Ca^2+^]mt in three groups. (**D**) Quantification of the [Ca^2+^]mt level (0 h n = 28; 6 h n = 25; 6 h + SAL n = 27). Scale bar = 20 μm. Results are presented as mean ± SEM. *** *p* < 0.001.

Calcium homeostasis of oocytes plays a significant role in the subsequent development capacity, Fluo 4-AM and Rhod 2-AM were used to detect the [Ca^2+^]c and [Ca^2+^]mt, respectively. The results showed that the [Ca^2+^]c (0 h vs. 6 h, *p* < 0.001; 6 h vs. 6 h + SAL, *p* < 0.001; Figure 4A,B) and [Ca^2+^]mt (0 h vs. 6 h, *p* < 0.001; 6 h vs. 6 h + SAL, *p* < 0.001; Figure 4C,D) were significantly increased in aged oocytes. As expected, SAL supplementation effectively restores the [Ca^2+^]c and [Ca^2+^]mt levels of aged oocytes.

### 3.5. SAL Inhibits DNA Damage, Early Apoptosis and Improves Spindle Assembly in Aged Oocytes

**Figure 5:**
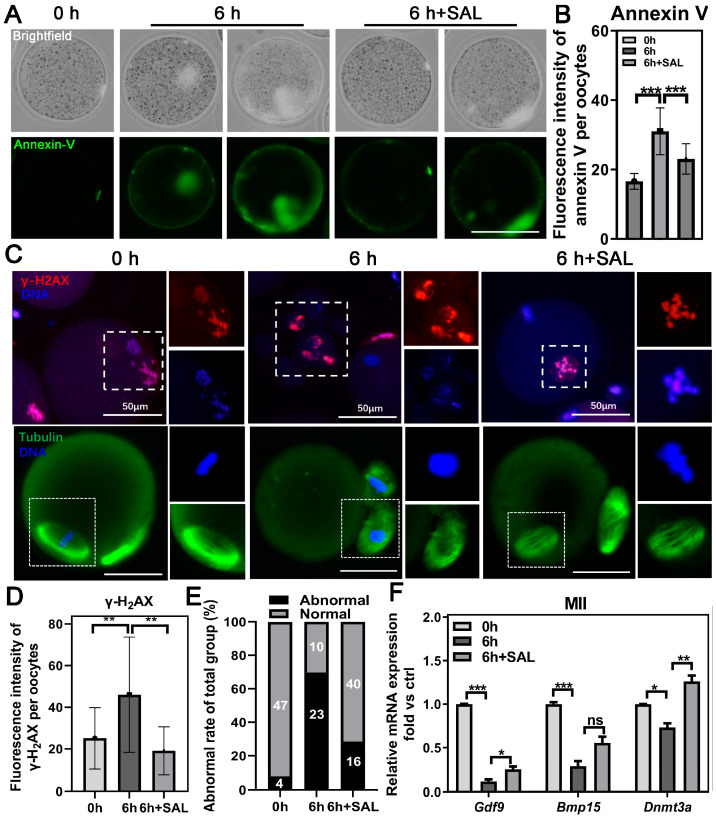
SAL can alleviate oxidative stress-induced cell damage. (**A**) Representative images of Annexin-V in young and aged oocytes with or without SAL administration. (**B**) Quantification of the Annexin-V level (0 h n = 39; 6 h n = 30; 6 h + SAL n = 35). (**C**) Representative images of DNA damage and meiotic spindle assembly in young and aged oocytes with or without SAL administration. (**D**) Relative fluorescence intensity of γH2A.X signals in three groups (0 h n = 47; 6 h n = 52; 6 h + SAL n = 46). (**E**) Comparison of abnormal spindle formation in three groups (0 h n = 51; 6 h n = 33; 6 h + SAL n = 56). (**F**) The mRNA expression of *Gdf9*, *Bmp15* and *Dnmt3a* in MII oocytes. Scale bar = 50 μm. Results are presented as mean ± SEM. ns *p* > 0.05; * *p* < 0.05; ** *p* < 0.01; *** *p* < 0.001.

Oxidative stress usually leads to the accumulation of DNA damage and accelerates early apoptosis. To evaluate the initiation of apoptosis in oocytes, we employed an Annexin-V probe. The addition of SAL effectively inhibited the augmented percentage of early apoptosis in aged oocytes (0 h vs. 6 h, *p* < 0.001; 6 h vs. 6 h + SAL, *p* < 0.001; Figure 5A,B). Meanwhile, we examined the extent of DNA damage using γ-H2A.X staining. The process of postovulatory aging resulted in a heightened fluorescence intensity of γ-H2A.X, which could be rescued by SAL treatment (0 h vs. 6 h, *p* < 0.01; 6 h vs. 6 h + SAL, *p* < 0.01; Figure 5C,D). Additionally, we detected spindle morphology and the results showed that postovulatory aging results in an increased rate of abnormal spindle apparatuses but SAL improved the formation of normal spindles (Figure 5C,E). The expression of genes associated with developmental potential, including *Gdf9*, *Bmp15* and *Dnmt3a*, were restored to some extent after SAL treatment (Figure 5F).

### 3.6. SAL Treatment Improves the Early Embryonic Development and Quality of Postovulatory Aged Oocytes

**Figure 6:**
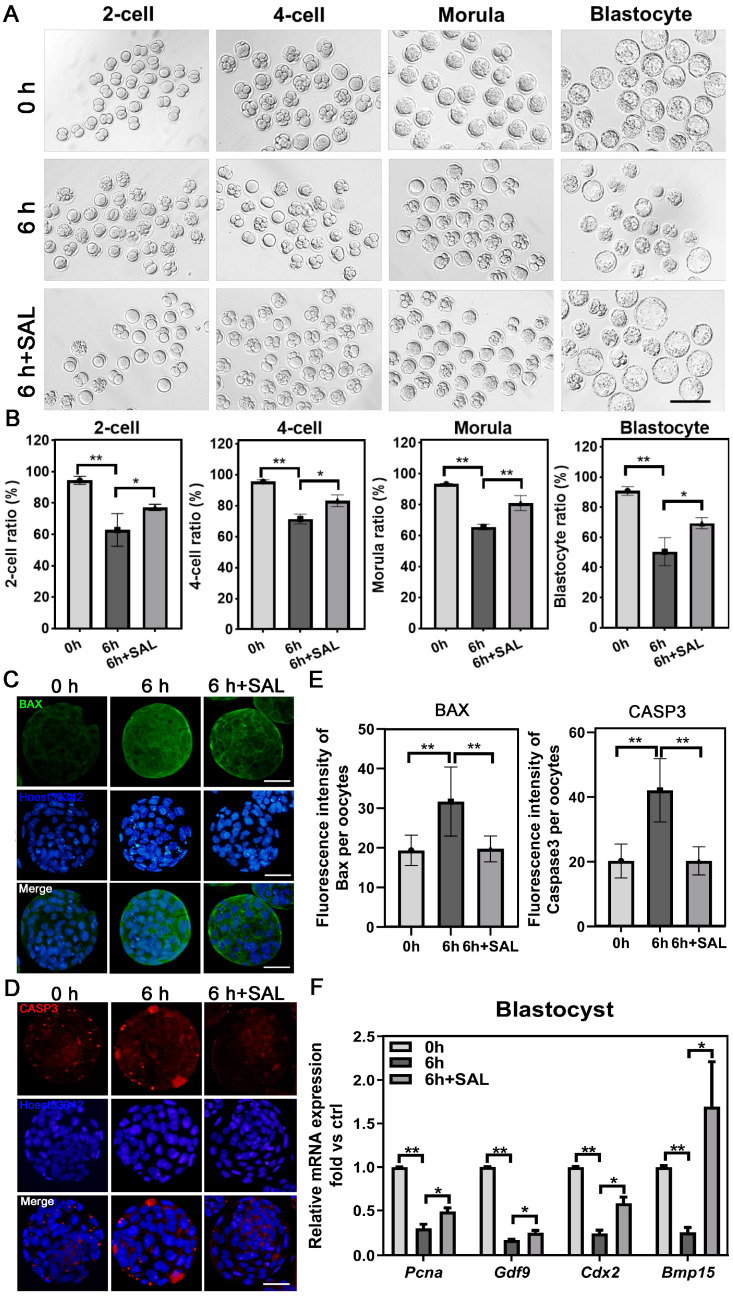
SAL promoted the early embryo development of postovulatory aged oocytes. (**A**) Representative images of early embryos developed from young and aged oocytes with or without SAL administration (scale bar = 100 μm). (**B**) The rate of 2-cell (0 h n = 39; 6 h n = 30; 6 h + SAL n = 35), 4-cell (0 h n = 39; 6 h n = 30; 6 h + SAL n = 35), morula (0 h n = 39; 6 h n = 30; 6 h + SAL n = 35) and blastocyte (0 h n = 39; 6 h n = 30; 6 h + SAL n = 35) in three groups. (**C**,**D**) Immunostaining of BAX and CASP3 in Ctrl, 6 h and 6 h + SAL groups (scale bar = 50 μm). (**E**) The fluorescence levels of BAX (0 h n = 29; 6 h n = 31; 6 h + SAL n = 33) and CASP3 (0 h n = 51; 6 h n = 73; 6 h + SAL n = 39) signals. (**F**) The mRNA expression levels of *Pcna*, *Gdf9*, *Cdx2* and *Bmp15* in blastocytes. Results are presented as mean ± SEM. * *p* < 0.05; ** *p* < 0.01.

Above, we confirmed that SAL enhances the quality of aged oocytes; thus, we also attempted to evaluate whether SAL improves early embryonic development and quality. In the young group, most MII ovulated oocytes can develop into 2-cell, 4-cell, morula and blastocytes. For 6 h aged oocytes, the proportions of all stage embryos decreased. However, in the SAL-supplemented aged oocyte group, the proportions of all stage embryos were significantly increased (Figure 6A,B and Table S3). In addition, we detected the embryo quality by IF and qPCR. The results showed that postovulatory aging increased the expression of BAX and CAS3 in embryos, which could be rescued by SAL treatment (BAX: 0 h vs. 6 h, *p* < 0.01; 6 h vs. 6 h + SAL, *p* < 0.01; CAS3: 0 h vs. 6 h, *p* < 0.01; 6 h vs. 6 h + SAL, *p* < 0.01, Figure 6C–E). In contrast, the mRNA expression of proliferation-related genes, including *Pcna*, *Gdf9*, *Cdx2* and *Bmp15*, declined in aged embryos but increased by SAL (Figure 6F). These results indicate that SAL treatment improves the early embryo development of aging oocytes and the quality of blastocysts.

### 3.7. RNA-Seq of Oocytes

**Figure 7:**
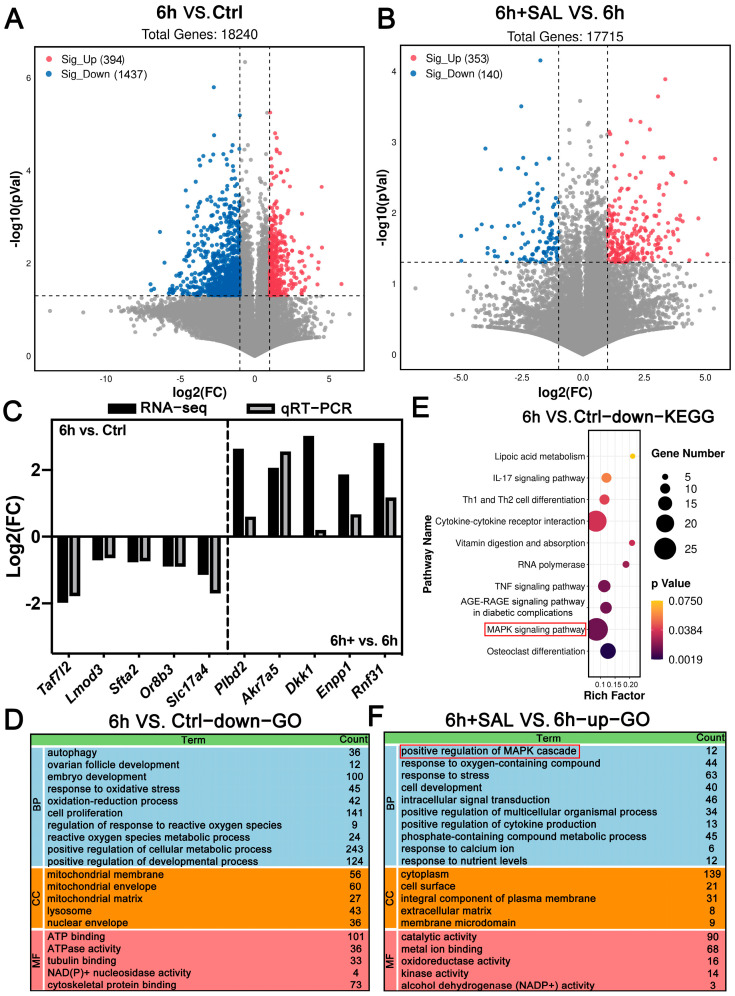
RNA-seq data shows gene expression changes in three groups. (**A**,**B**) Volcano plots show DEGs from 6 h vs. Ctrl group and 6 h + SAL vs. 6 h group. (**C**) Verification of expression levels of DEGs by qRT-PCR. (**D**) GO functional annotation of DEGs from 6 h vs. Ctrl group for biological processes (BP), cellular components (CC) and molecular functions (MF). (**E**) KEGG pathway analysis of DEGs from 6 h vs. Ctrl group. (**F**) GO functional annotation of DEGs from 6 h + SAL vs. 6 h group.

To delve deeper into the latent mechanism behind the enhanced quality of aged oocytes after SAL treatment, we analyzed the transcriptome profiles of oocytes from three groups using RNA-seq. A total of 1831 and 493 DEGs were found in the comparison of 6 h vs. Ctrl and 6 h + SAL vs. 6 h, respectively (Figure 7A,B and Table S2). The qRT-PCR was utilized to confirm the authenticity of the RNA-seq data by analyzing randomly chosen genes (Figure 7C).

Subsequently, GO term and KEGG enrichment analysis conducted an in-depth investigation of the differentially expressed genes (DEGs). After 6 h aging, the downregulated gene-GO enrichment analysis revealed that postovulatory aging damages autophagy, ovarian follicle development, cell proliferation, ROS metabolic process and mitochondrial membrane, etc. (Figure 7D). The KEGG analysis manifested that the MAPK pathway was suppressed in aged oocytes (Figure 7E). Additionally, we observed that the MAPK cascade was activated in oocytes after SAL treatment, meanwhile, the ability of the stress response was enhanced (Figure 7F). The above results suggested that postovulatory aging leads to mitochondrial dysfunction and the decrease in autophagy in oocytes, and the MAPK signaling pathway plays an important role in the aged process and SAL effects.

### 3.8. SAL Promotes Protective Autophagy of Aged Oocytes by Stimulating the MAPK Signaling Pathway

**Figure 8:**
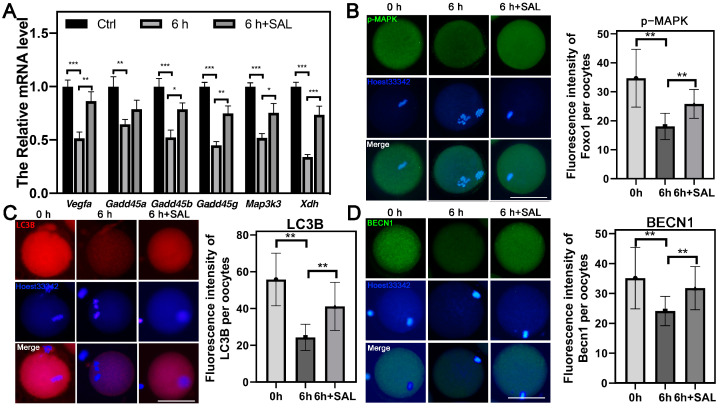
SAL promotes protective autophagy of aged oocytes by stimulating the MAPK signaling pathway. (**A**) The relative mRNA expression of *Vegfa*, *Gadd45a*, *Gadd45b*, *Gadd45g*, *Map3k3* and *Xdh* in Ctrl, 6 h and 6 h + SAL groups. (**B**) Immunostaining of p-MAPK in Ctrl, 6 h and 6 h + SAL groups and the fluorescence levels of p-MAPK signals (0 h n = 41; 6 h n = 38; 6 h + SAL n = 41). (**C**) Immunostaining of LC3B in Ctrl, 6 h and 6 h + SAL groups and the fluorescence level of LC3B signals (0 h n = 38; 6 h n = 39; 6 h + SAL n = 37). (**D**) Immunostaining of BECN1 in Ctrl, 6 h and 6 h + SAL groups and the fluorescence level of BECN1 signals (0 h n = 30; 6 h n = 40; 6 h + SAL n = 41). Scale bars = 50 μm. Results are presented as mean ± SEM. * *p* < 0.05; ** *p* < 0.01; *** *p* < 0.001.

Based on the transcriptome data, we tested the mRNA expression of different genes in the MAPK signaling pathway and the variation trends were similar to the results of RNA-seq (Figure 8A). Simultaneously, the IF result confirmed that the p-MAPK was significantly increased after SAL treatment (0 h vs. 6 h, *p* < 0.01; 6 h vs. 6 h + SAL, *p* < 0.01, Figure 8B). In addition, we detected the autophagy level of oocytes by testing the expression of autophagy-related proteins including LC3B and BECN1. The expression levels of two proteins in aged oocytes were significantly lower than those in the ovulated oocytes, while SAL treatment made them recover (LC3B: 0 h vs. 6 h, *p* < 0.01; 6 h vs. 6 h + SAL, *p* < 0.01; BECN1: 0 h vs. 6 h, *p* < 0.01; 6 h vs. 6 h + SAL, *p* < 0.01, Figure 8C,D). These results demonstrated that SAL treatment reversed the inhibition of protective autophagy in aged oocytes by stimulating the MAPK pathway.

## 4. Discussion

The process of human-assisted reproductive technology (ART) and livestock-timed insemination faces challenges due to the problem of oocyte postovulatory aging. This issue often leads to failure in IVF or ICSI procedures and breeding. To address this problem, we propose the use of SAL, a natural plant antioxidant, to improve the quality of postovulatory aged oocytes. To investigate this hypothesis and explore the underlying mechanisms thoroughly, we employed an experimental model using in vitro aged mouse oocytes after ovulation. Our goal is to establish a more robust foundation for the potential application of SAL in both human ART and livestock breeding.

In this study, we initially assessed the impact of postovulatory aging on the structure and integrity of mouse oocytes. Previous research has consistently demonstrated that cellular fragmentation, which is an important measure of oocyte quality, typically occurs in older oocytes, resulting in a decrease in their ability to develop [14]. Consistent with these previous findings, we observed a substantial increase in cellular fragmentation in oocytes as the aging period extended, although there were no significant variations across the four time-intervals. However, the addition of SAL resulted in a reduction in the rate of fragmentation at various time intervals and concentrations, suggesting that SAL possesses the potential to enhance oocyte quality. In order to further elucidate the mechanisms by which SAL ameliorates the decline in oocyte competence induced by aging, we subsequently investigated the associated indicators of oocyte quality in postovulatory aged oocytes.

Previous research has reported that postovulatory aging could induce the generation of excessive ROS leading to concomitantly decreased antioxidant protection [25]. Here, we observed that ROS accumulation was significantly reduced when oocytes were treated with SAL. Consistent with this finding, BEAS-2B cells treated with SAL exhibited decreased PM2.5-induced intracellular ROS levels [26]. Additionally, GSH, which is the abundant antioxidant in cells, influences the maturation of the cytoplasm. This is associated with the fertilization of oocytes and the subsequent progression [27]. Furthermore, studies have indicated a decrease in the GSH concentration in postovulatory aged mouse oocytes [28]. Similar to previous studies, we observed that SAL significantly increased GSH contents within in vitro aged oocytes, suggesting that SAL exerts antioxidant effects in the postovulatory aging process. Additionally, we detected the mRNA expression of antioxidant enzymes. Frontline mediators of redox equilibrium include ROS scavenging enzymes such as SOD2 and GPx1 [29]. SOD2 has been linked to the prevention of segregation defects during oocyte aging [30], while GPx enhances the developmental capacity of oocytes and embryos [31]. Consequently, the increased expression of these antioxidant enzymes is crucial for safeguarding oocytes against oxidative stress resulting from postovulatory aging. In our investigation, we validated the reduction in expression levels of antioxidant enzyme genes (*Sod2* and *Gpx1*) under in vitro aging conditions, which were subsequently restored following SAL treatment.

Mitochondria play a crucial role in the development of oocytes, as it is responsible for producing the ATP in oocytes and early embryos [32]. Mitochondrial dysfunction is strongly associated with a decrease in chromosomal segregation disorders, failures in fertilization, oocyte fragmentation, and apoptosis driven by mitochondria [33]. It is reported that mitochondrial distribution and function can change during the process of postovulatory aging [34,35,36]. Therefore, we conducted further investigations to determine how SAL affects the structure and function of mitochondria in aged oocytes. Consistent with their results, our findings uncovered that SAL safeguards mitochondrial function by restoring normal patterns of mitochondrial distribution and increasing the mitochondrial membrane potential (ΔΨm). Additionally, the ATP content in aged oocytes enhanced significantly after SAL treatment. Moreover, mitochondria additionally possess numerous functions, encompassing the control of calcium and actively engaging in the regulation of pathways for transmitting signals [37], meanwhile, disequilibrium of calcium homeostasis causes mitochondrial dysfunction likewise [38]. According to reports, Ca^2+^ release in oocytes, induced by postovulatory aging, caused abnormally elevated levels of Ca^2+^ in mice and rats [39,40,41]. Our findings further verified this phenomenon. The findings support the notion that SAL has the potential to restore intracellular calcium homeostasis and mitochondrial function.

While aged oocytes have the capability to expel the initial polar body following the resumption of meiosis, there is a higher likelihood of errors occurring in chromosome alignment and spindle formation in both experimental animals and humans [42,43,44,45]. Our experiment results found that the abnormal spindle proportion in aged oocytes was significantly higher than in ovulated oocytes, whereas SAL treatment alleviated this increase. The effect of SAL on the spindle is similar to that of putrescine and quercetin [25,46], while resveratrol is not as effective [47]. Probably, a low dosage and short-term treatment of resveratrol may not be sufficient to rescue the spindle aberrations in aging oocytes. Additionally, increasing studies confirmed that oxidative stress associated with aging is the main reason for the deterioration of oocyte quality [48], which triggers a series of cascades that influence oocyte development, including oocyte apoptosis and DNA damage [24,49,50]. Our findings illustrated that SAL was able to reduce the aging-induced DNA damage, thereby suppressing oocyte apoptosis. Meanwhile, the qPCR result showed that the expression of proliferation-related genes was increased after SAL treatment.

Postovulatory aging can cause multiple biochemical and functional alterations in oocytes damaging the early embryonic development including the decline of cleaved and blastocyst rate [24,25,51]. In view of the deterioration of various related indicators of aged oocytes, we detected the early embryonic development of aged oocytes by parthenogenesis activation. As is expected, the developmental levels in all stages of aged oocytes significantly declined. The embryonic degeneration of aging oocytes manifests in several forms, including development arrest, embryo irregular division, and fragmentation formation, which would damage embryo development and implantation [52]. Similarly, we evaluated the quality of blastocysts from different treatment oocytes and found that SAL treatment significantly improved their apoptosis and proliferation levels.

In order to further explore the mechanism of SAL treatment improving the quality of aging oocytes, we analyzed the transcriptome of oocytes. The analysis results suggested that the mitochondrial functions of oocytes were damaged and the autophagic function inhibited after 6 h aging. On the one hand, mitochondria affect all aspects of mammalian reproduction [53]. Normal mitochondrial function is essential for optimal oocyte maturation, fertilization and embryonic development [54,55]. It was reported that mitochondrial dysfunction causes a decrease in oocyte quality of aged mice and interferes with embryonic development [56,57]. This result is consistent with that obtained in our experiment. On the other hand, autophagy, a process of self-eating of cellular materials, is requisite to reprocess the damaged cell organelles to procure new building blocks for maintaining cellular homeostasis [58] and is involved in reproductive function including granulosa cell proliferation, follicle development, and oocyte maturation [59]. Moreover, autophagy is obligatory for cell survival during stress conditions [60]; however, autophagy was inhibited in aged oocytes in our study. Nevertheless, the autophagic function of aged oocytes was recovered by treatment with SAL. In addition, the MAPK signaling pathway is crucial in SAL action. Our result is similar to previous research that the MAPK signaling pathway could induce cell autophagy [61,62]. In pigs, the supplementation of SAL in maturation medium in vitro not only enhanced the subsequent blastocyst formation rate and embryo quality but also improved the redox state and mitochondrial function [11,12]. Meanwhile, one of the studies found that the mRNA expressions of the MAPK pathway and MAPK phosphorylation in oocytes increased significantly in the SAL group [11]. Finally, we conducted a verification experiment and found that the MAPK signaling pathway was activated and stimulated protective autophagy in aged oocytes after SAL treatment. Interestingly enough, a recent study has shown that spermidine-induced recovery of oocyte quality was mediated by enhancement of mitophagy activity and mitochondrial function in aged mice [63]. This research once again demonstrated the significant role of autophagy in the development of age-related oocytes. However, the effect evaluation of SAL on aged oocytes was only performed in mice model in vitro, thus, the effect on in vivo experiments and ART needs further study.

## 5. Conclusions

**Figure 9:**
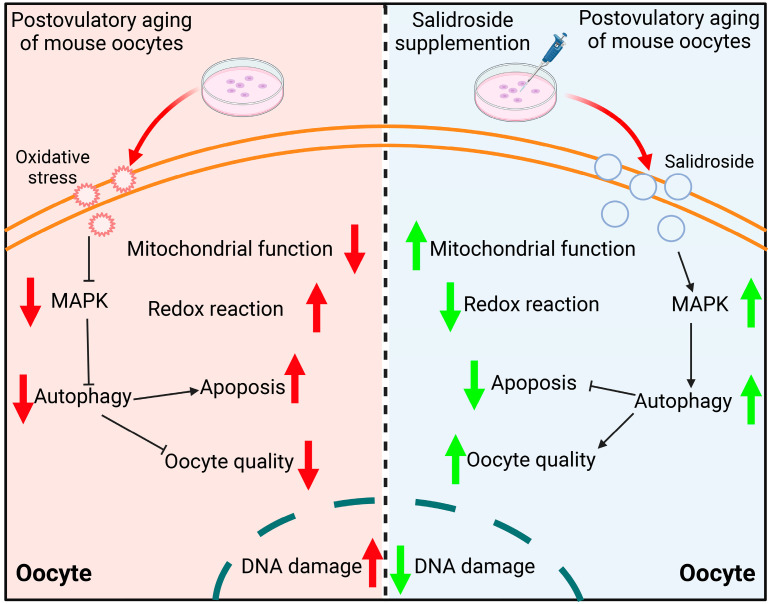
Schematic model illustrating the potential mechanism associated with salidroside-induced increase in quality and autophagy in aged oocytes. The red and green arrows indicated the rise and fall of biological processes or some indels.

Long-term exposure in the no-sperm environment results in a decrease in the quality of the ovulated oocytes. In this study, we provided that SAL moderated oxidative stress, alleviated mitochondrial dysfunction and promoted protective autophagy by stimulating the MAPK pathway, which improved fertilization and embryonic development (Figure 9). Therefore, we believe that SAL may be a new and effective natural compound substance for delaying the aging process of oocytes.

## Figures and Tables

**Table 1:** Primer sequences for qRT-PCR.

Gene	Primer Sequence	Accession Numbers	Product Length (bp)
*Gpx1*	F: AGTCCACCGTGTATGCCTTCT	NM_008160.6	105
	R: GAGACGCGACATTCTCAATGA		
*Sod2*	F: CAGACCTGCCTTACGACTATGG	NM_013671.3	113
	R: CTCGGTGGCGTTGAGATTGTT		
*Gdf9*	F: TCTTAGTAGCCTTAGCTCTCAGG	NM_008110.2	116
	R: TGTCAGTCCCATCTACAGGCA		
*Bmp15*	F: TCCTTGCTGACGACCCTACAT	NM_009757.5	100
	R: TACCTCAGGGGATAGCCTTGG		
*Dnmt3a*	F: GAGGGAACTGAGACCCCAC	NM_001271753.2	216
	R: CTGGAAGGTGAGTCTTGGCA		
*Pcna*	F: TTTGAGGCACGCCTGATCC	NM_011045.2	135
	R: GGAGACGTGAGACGAGTCCAT		
*Cdx2*	F: CAAGGACGTGAGCATGTATCC	NM_007673.3	106
	R: GTAACCACCGTAGTCCGGGTA		
*Taf7l2*	F: ATGAGCAAAAGCCGAGATGAA	NM_001161855.1	108
	R: ATTTCCAGAACGGATGATCTTCC		
*Lmod3*	F: ACTCCTAGCCAACTTATCCCC	NM_001081157.2	214
	R: ACTGACAGGAACTCGTTCATCT		
*Sfta2*	F: AGCTGACCGAGACTTTTCAGG	NM_001163194.1	131
	R: GTGTGGTGGTCCTTTGTGAAG		
*Or8b3*	F: TCTTTTGCTGCGTATGGGATG	NM_146869.2	135
	R: GCAGGAAAGTTGAAGGAGAGGA		
*Slc17a4*	F: ACTGGAGCAGACCTGAAAGC	NM_177016.3	160
	R: AGCTCAAGTTCATTTGTTGGGTA		
*Vegfa*	F: CTGCCGTCCGATTGAGACC	NM_001025250.3	233
	R: CCCCTCCTTGTACCACTGTC		
*Gadd45a*	F: CCGAAAGGATGGACACGGTG	NM_007836.1	121
	R: TTATCGGGGTCTACGTTGAGC		
*Gadd45b*	F: CAACGCGGTTCAGAAGATGC	NM_008655.1	122
	R: GGTCCACATTCATCAGTTTGGC		
*Gadd45g*	F: GGGAAAGCACTGCACGAACT	NM_011817.2	119
	R: AGCACGCAAAAGGTCACATTG		
*Map3k3*	F: ATAAGGACACAGGTCACCCAA	NM_011947.4	115
	R: TGCTCCACATCTTCGTATCTCA		
*Xdh*	F: ATGACGAGGACAACGGTAGAT	NM_011723.3	185
	R: TCATACTTGGAGATCATCACGGT		
*Plbd2*	F: CAACATCCCGTACTTTGAGACTG	NM_023625.4	229
	R: CTCCGCATTAGGCTTTGGGTT		
*Akr7a5*	F: CGGCCAGTCCGAGAACATC	NM_025337.3	108
	R: TTCAGTGACTTCCCTTCCCAG		
*Dkk1*	F: CTCATCAATTCCAACGCGATCA	NM_010051.3	105
	R: GCCCTCATAGAGAACTCCCG		
*Enpp1*	F: CTGGTTTTGTCAGTATGTGTGCT	NM_001308327.1	231
	R: CTCACCGCACCTGAATTTGTT		
*Rnf31*	F: GCCCTGAGGTGGGATTCTG	NM_194346.3	152
	R: TTGAGGTAGTTTCGAGGCTCC		
*Actin*	F: GATGCCCCCACCGACTTTATC	NM_007393	154
	R: CCAGTTGGTAACAATGCCATGT		

## Data Availability

All the data are presented in the article.

## Supplementary Materials

The following supporting information can be downloaded at: https://www.mdpi.com/article/10.3390/antiox13020248/s1, Table S1: SAL Decreases Oocyte Malformation Rate During Postovulatory Aging; Table S2: DEGs in oocyte transcriptome in the comparison of 6 h vs. Ctrl and 6 h + SAL vs. 6 h; Table S3: SAL Improves the Early Embryonic Development During Postovulatory Aging.
