# Supplementary material for: The Antioxidant Salidroside Ameliorates the Quality of Postovulatory Aged Oocyte and Embryo Development in Mice
Source: Antioxidants (Basel). 2024 Feb 19;13(2):248. doi: 10.3390/antiox13020248 (PMC10886307; doi:10.3390/antiox13020248)
Supplement: Supplementary file 1 [file antioxidants-13-00248-s001.zip › Tables S1 and S3.pdf]

Table S1. SAL Decreases Oocyte Malformation Rate During Postovulatory Aging

Treated Time	0μm SAL (%)	10μm SAL (%)	20μm SAL (%)	40μm SAL (%)
6 h	39.8±1.72 (n=87)	16.0±4.48 (n=85)	12.0±0.83 (n=102)	26.1±4.82 (n=104)
12 h	43.1±6.32 (n=80)	11.8±2.55 (n=82)	22.5±2.88 (n=95)	21.7±2.82 (n=124)
18 h	43.3±1.62 (n=133)	9.72±1.57 (n=120)	21.1±1.08 (n=81)	22.7±6.30 (n=86)
24 h	49.3±1.71 (n=108)	13.7±0.32 (n=66)	11.3±1.31 (n=71)	17.8±3.14 (n=69)

Table S3. SAL Improves the Early Embryonic Development During Postovulatory Aging

Rate (%)	0 h	6 h	6 h+SAL
2-cell	94.52±1.469	62.87±5.981	77.28±1.160
4-cell	95.63±0.671	71.42±1.837	83.31±2.153
Morula	93.49±0.166	65.66±1.01	81.08±2.782
Blastocyte	90.75±1.467	50.46±4.684	69.35±2.155
